# 751. The success of an escape room activity on antimicrobial stewardship in achieving Doctor of Pharmacy Educational outcomes

**DOI:** 10.1093/ofid/ofad500.812

**Published:** 2023-11-27

**Authors:** Yuman Lee, Nicole Bradley

**Affiliations:** St. John's University, Queens, New York; St. John's University, Queens, New York

## Abstract

**Background:**

ASP Escape Room was an activity created and conducted in the infectious disease elective for pharmacy students in their 3rd professional year. Students were assigned the CDC Core Elements of ASP as a pre-reading. During class, students worked in groups of 4 on tasks relating to the CDC Core Elements to decode various puzzles to advance through the activity in establishing an antimicrobial stewardship program. The goal of ASP Escape Room was to engage and motivate students to learn foundational antimicrobial stewardship concepts and challenge students to develop personally and professionally. The purpose of this study was to assess the impact of ASP Escape Room on achieving Doctor of Pharmacy Curriculum Outcomes and Entrustable Professional Activities (COEPA) 2022 and evaluate perceptions.

**Methods:**

During the Spring 2023 semester, student feedback was anonymously collected via an electronic survey. Students evaluated the impact of ASP Escape Room based on the 3 domains of COEPA 2022 (Knowledge, Skills, and Attitudes) on a Likert scale from strongly disagree to strongly agree.

**Results:**

39 students responded. Majority strongly agreed or agreed that ASP Escape Room allowed them to learn foundational knowledge (38/39, 97.4%), develop problem solving skills (37/39, 94.9%), improve effective communication skills (38/39, 97.4%), understand the role of a steward in optimizing healthcare (39/39, 100%), achieve shared goals on a team (38/39, 97.4%), and build/maintain trust among colleagues (39/39, 100%). Additionally, students enjoyed participating (38/39, 97.4%), reported the activity was an engaging way of learning antimicrobial stewardship (38/39, 97.4%), and that they would like to participate in future escape room activities as part of pharmacy course work (36/39, 92.3%). Most students ranked the activity’s difficulty as a 3 (61.5%) or 4 (30.8%) out of 5.

**Conclusion:**

ASP Escape Room was positively received and provided an opportunity for students to expand beyond foundational knowledge, and develop on the and skills and attitudes domains, including building trust, effective communication, and supporting team goals. Opportunities like ASP Escape Room Activity are important in the education of pharmacy students to prepare them to practice at the highest level of the profession.

**Disclosures:**

**All Authors**: No reported disclosures

